# Congenital Malformations of the Eye: A Pictorial Review and Clinico-Radiological Correlations

**DOI:** 10.1155/2024/5993083

**Published:** 2024-01-30

**Authors:** Alessia Guarnera, Paola Valente, Luca Pasquini, Giulia Moltoni, Francesco Randisi, Chiara Carducci, Alessia Carboni, Giulia Lucignani, Antonio Napolitano, Antonino Romanzo, Daniela Longo, Carlo Gandolfo, Maria Camilla Rossi-Espagnet

**Affiliations:** ^1^Neuroradiology Unit, Imaging Department, Bambino Gesù Children's Hospital, IRCCS, Piazza S. Onofrio 4, Rome 00165, Italy; ^2^Neuroradiology Unit, NESMOS Department, Sant'Andrea Hospital, La Sapienza University, Via di Grottarossa 1035-1039, Rome 00189, Italy; ^3^Ophthalmology Unit, Bambino Gesù Children's Hospital, IRCCS, Piazza S. Onofrio 4, Rome 00165, Italy; ^4^Neuroradiology Service, Department of Radiology, Memorial Sloan Kettering Cancer Center, New York 10065, NY, USA; ^5^Medical Physics Department, Bambino Gesù Children's Hospital, Rome, Italy

## Abstract

Congenital malformations of the eye represent a wide and heterogeneous spectrum of abnormalities that may be part of a complex syndrome or be isolated. Ocular malformation severity depends on the timing of the causative event during eye formation, ranging from the complete absence of the eye if injury occurs during the first weeks of gestation, to subtle abnormalities if the cause occurs later on. Knowledge of ocular malformations is crucial to performing a tailored imaging protocol and correctly reporting imaging findings. Together with the ophthalmologic evaluation, imaging may help frame ocular malformations and identify underlying genetic conditions. The purpose of this pictorial review is to describe the imaging features of the main ocular malformations and the related ophthalmologic findings in order to provide a clinico-radiological overview of these abnormalities to the clinical radiologist. Sight is a crucial sense for children to explore the world and relate with their parents from birth. Vision impairment or even blindness secondary to ocular malformations deeply affects children's growth and quality of life.

## 1. Introduction

Ocular malformations represent a wide range of ocular abnormalities, with an incidence ranging from 0.36% to 4.7% [1–5]. Congenital ocular abnormalities may be isolated or syndromic, and the prevalence is higher in premature and stillborn children [4–7].

Vision impairment secondary to ocular malformation deeply affects patients' quality of life and leads to massive neuroanatomical plasticity, affecting brain development [1, 8].

This review aims to describe the main ocular malformations and their typical imaging and clinical findings to promote a multidisciplinary vision of these disorders.

## 2. Eye Embryology

Eye embryological development spans across embryonic and fetal stages [7] and continues after birth, with posterior segment expansion accounting for over 90% of postnatal growth [9]. Eye development starts at approximately day 22 of gestation when the primary optic vesicle arises from the diencephalic forebrain wall [10, 11] and subsequently enlarges and invaginates into the secondary optic vesicle [10]. This one consists of two layers: the internal retinal pigmented layer and the external retinal nervous layer [7, 11]. At the beginning of the fourth week, the optic vesicle induces the surface ectoderm to form the lens placode [11], which transits to the lens vesicle by the 5^th^ week of gestation [10]. At the same time, the choroid and sclera originate from the surrounding mesenchyme, while the vitreous body originates from the mesenchyme located in the space between the lens vesicle and the secondary optic vesicle [10]. By the 35^th^ week of gestation, the primitive vascular system, forming the primary vitreous and consisting of the hyaloid artery and its branches, is involved, and the secondary vitreous takes its place [7]. Development and differentiation of the retinal nervous layer last until the 8^th^ month of gestation, when the eye becomes sensitive to light and axons from the retinal neurons extend to the brain, forming the optic nerve. During the whole embryological process, several genetic factors play a fundamental role in orchestrating eye development, and both genetic mutations and clastic events may influence embryological development.

## 3. MRI Anatomy of the Ocular Globe

The patient is usually referred to the ophthalmologist by the general paediatrician in relation to any abnormality observed at clinical examination. Ocular malformations may be diagnosed or suspected by the ophthalmologist, who may require further diagnostic imaging investigations. US is a noninvasive, cost-limited, and radiation-free first-level radiological exam that shows the anatomy and pathology of the ocular globe. MRI is a second-level radiological examination that allows a panoramic evaluation of the ocular globe, the surrounding tissues, and the brain. It is crucial to investigate a particular malformation of the eye and the suspicion of a complex syndrome involving the eye, surrounding structures, and/or the brain. The MRI exam consists of some specific sequences acquired in different planes and characterized by different radiological parameters which may highlight peculiar structures or tissues. The main sequences of an MRI orbital protocol are T1WI and T2WI, which allow us to identify the anatomy and the pathology of the orbit and the ocular globe. T1WI are key sequences to define the morphology of the globe and are particularly sensitive to the presence of blood. After gadolinium administration, T1WI may show the physiological and pathological enhancement of the eye structures. Highly vascularized tissues appear hyperintense after gadolinium administration, as well as some pathological neoformations. T2WI sequences are pivotal for the analysis of the ocular globe, which appears mainly hyperintense on T2WI since highly hydrated tissues, and fluids such as the vitreous and aqueous humors, appear hyperintense on T2WI. Pathological conditions in which there is a reduction of hydrated tissues result in signal modifications [12–18].

**Figure 1:**
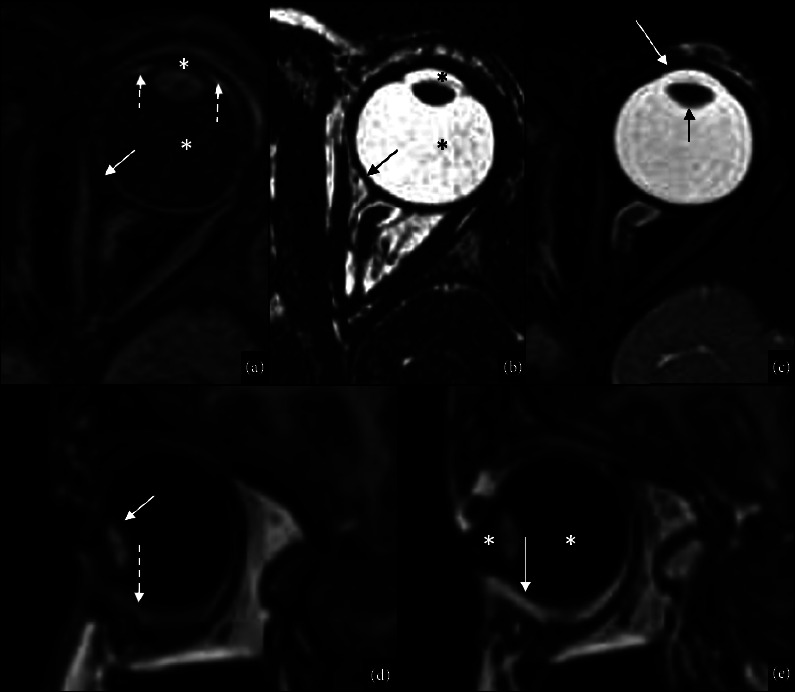
Radiological anatomy of the ocular globe. Radiological anatomy of the ocular globe. MRI axial postcontrast T1WI (a), DRIVE (b), T2WI (c), sagittal precontrast T1 (d), and postcontrast T1 (e). The predominant MR signal of the ocular globe is related to aqueous and vitreous humors appearing hyperintense on T2WI/DRIVE (black asterisks in (b)) and hypointense on T1WI (white asterisks in (a) and (e)). The external layer is composed of the sclera and the cornea, appearing hypointense on both T1WI and T2WI (sclera: white arrow in (a) and black arrows in (b); cornea: white arrow in (c)). The middle layer corresponds to the uvea, which is composed of the iris, the ciliary body, and the choroid. The uvea appears hyperintense on T1WI (dotted white arrow in (d)) and hypointense on T2WI. After contrast administration, the uvea, in particular, the choroid (white arrow in (e)) and the ciliary body/iris (dotted white arrow in (a)), present a vivid enhancement. The inner layer corresponds to the retina, which cannot be told apart from the choroid, as it appears hypointense on T2WI and shows enhancement after contrast administration (white arrow in (e)). Crystalline lens is typically hypointense on T2WI (black arrow in (c)) and hyperintense on T1W (white arrow in (d)).

A comprehensive knowledge of radiological orbital anatomy together with a tailored MRI orbital protocol is crucial to studying orbital anatomy and related disorders (Table 1, Figure 1).

### 3.1. External Layer

The sclera and the cornea are made of collagen and therefore appear hypointense on both T1WI (weighted image) and T2WI. The cornea may be highlighted by an overlying slightly hyperintense tear film on T1WI [12].

### 3.2. Middle Layer

The uvea appears hyperintense on T1WI and hypointense on T2WI. After contrast administration, the uvea, particularly the choroid and the ciliary body, presents a vivid enhancement [12, 18].

### 3.3. Inner Layer

The retina cannot be distinguished from close structures in normal conditions, in particular from the choroid. It appears hypointense on T2WI and after contrast administration, it shows enhancement [12, 18].

### 3.4. Lens

Crystalline Lens is typically hypointense on T2WI and hyperintense on T1WI in relation to high structural protein content [12, 18].

### 3.5. Ocular Globe Humors

The predominant MR signal from the globe is from aqueous and vitreous humor which are hyperintense on T2WI and hypointense on T1WI because they have a >98% water content [12, 18].

## 4. Ocular Malformations

The congenital ocular malformation spectrum includes a variety of different abnormalities. The main features of each disease are presented in Table 2.

## 5. MAC Complex

The term “MAC complex” refers to the malformation spectrum of microphthalmia, anophthalmia, and coloboma. All these malformations are characterized by a smaller or absent ocular globe or absence of eye tissues. In both syndromic and nonsyndromic conditions, different genetic malformations are associated with the MAC complex.

### 5.1. Anophthalmia

**Figure 2:**
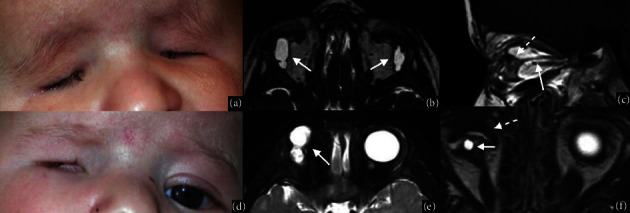
Anophthalmia and severe microphthalmia. Clinical bilateral anophthalmia is noted in a one-year-old boy (a–c). Axial high-resolution T2WI demonstrates bilateral anophthalmia with the presence of cysts (white arrows in (b)). On sagittal T1WI amorphous tissue (dotted white arrow in (c)) and linear structure consistent with remnants of the optic nerve sheaths are noted (white arrow in (c)). One-year-old girl with suspected right anophthalmia (d–f). Axial fat-suppressed T2WI shows an intraorbital cyst (white arrow in (e)) and axial DRIVE sequence (f) demonstrates the presence of a small ocular globe (white arrow in (f)) behind the prosthesis (dotted white arrow in (f)).

Anophthalmia is defined as the complete absence of ocular tissue in the eye socket that leads to complete blindness and can be associated with the absence of optic nerve, and hypoplastic optic chiasm [10]. Anophthalmia is extremely rare, having a prevalence of 3 per 100.000 live births [19, 20], and represents a continuum spectrum with microphthalmia. In complex cases characterized by extreme microphthalmia, it can be extremely difficult to identify an ocular globe at clinical examination; in these cases, the differential diagnosis between the two entities is made through a histological examination [6, 21]. However, a tailored MRI may be of help in identifying the extreme microphthalmic globe. The exact pathogenesis remains unknown, but a comprehensive medical and familiar history, physical examination, genetic testing, and imaging may help to establish a possible, specific cause [7, 11]. Three main types of anophthalmia have been identified: primary anophthalmia, which refers to the failure to outgrow the primary optic vesicle; secondary anophthalmia when there is the failure of development of the anterior portion of the neural tube; and tertiary or degenerative anophthalmia, in which an early injury occurred during ocular globe formation [6, 10, 21]. Some authors only identify two types of anophthalmia: primary anophthalmia, resulting from the failure of the optic pit to form a vesicle; and secondary anophthalmia, when the optic vesicle forms and degenerates [7, 11]. Primary anophthalmia may be unilateral or bilateral, occurring between the fourth and the seventh week of gestation [6], and has been speculated to be the result of mutations of genes responsible for optic vesicle genesis [11] and be part of various syndromes. The most involved gene is SOX2, mutations of which have been shown to account for 10% to 20% of cases of severe and bilateral anophthalmia/microphthalmia [11, 22]. Features associated with these mutations are oesophagal atresia, vertebral segmentation anomalies, facial dysmorphisms, and male genitalia anomalies [11, 21, 23]. Primary anophthalmia has also been associated with mutations of OTX and RAX [23, 24]. No brain anomalies have been related to these mutations [25, 26]. Other less frequently involved genes are CHX10, FOXE3, and PAX6 [21, 27]. Many chromosome anomalies have been related to anophthalmia, the most common being trisomy 13 (Patau syndrome) and trisomy 18 (Edwards syndrome) [21]. Among syndromes related to anophthalmia, we cite Matthew-Wood syndrome and De Morsier's syndrome [6]. Degenerative anophthalmia generally depends on an injury that occurred within the 8^th^ week of gestation [10] and may be secondary to infections (TORCH-toxoplasmosis, other, rubella, cytomegalovirus, herpes), vascular events, toxic and metabolic events (hypervitaminosis or hypovitaminosis A), fever and hyperthermia, X-rays exposure, drugs exposure as thalidomide and warfarin, and exposure to toxins as alcohol, solvents, and pesticides [6, 10, 11, 21, 28–30]. Trauma may be considered either a potential cause of degenerative anophthalmia or a fourth type of anophthalmia, especially when referring to direct trauma to the globe [6]. Other maternal and child factors may increase the risk of anophthalmia such as maternal age over 40 and multiple births for maternal factors or low birth weight and premature birth as child factors [6]. Patients with anophthalmia present with monocular vision if anophthalmia is unilateral and blindness if anophthalmia is bilateral. Anophthalmia may be investigated pre- and post-natally with US and MRI. US shows the nonvisualization of the eyeball and lens, while MRI detects the absence of the eyeball and the presence of amorphous, T1WI isointense, and T2WI hyperintense tissue in the ocular socket (Figures 2(a)–2(c)).

Differential diagnoses are cryptophthalmos, which refers to wholly fused eyelid margins, without lashes; cyclopia, and synophthalmia, which, respectively, refer to the total and partial fusion of optic vesicles [21]. The optimal treatment should begin early after birth and consists of a simultaneous expansion of the eyelids, socket, and orbital bones. Socket expansion may be obtained through self-inflating expanders and custom-made conformers which progressively increase in size [31]. Orbital osteotomies are indicated in more severe cases [32]. Following socket expansion, dermis-fat grafts, and orbital implants are adequate options [31].

### 5.2. Microphthalmia

**Figure 3:**
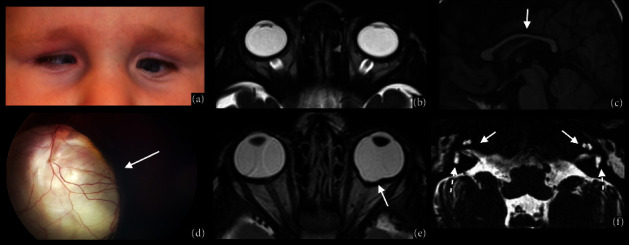
Simplex microphthalmia and complex colobomatous microphthalmia. Bilateral congenital microphthalmia in a one-year-old boy affected by 16q22 deletion syndrome (a–c). MRI T2WI shows bilateral primary microphthalmos, characterized by reduced anteroposterior and transverse ocular globe diameters (b). Sagittal T1WI of the brain shows associated corpus callosum hypoplasia (white arrow in (c)). RET-cam image of the left eye shows microphthalmia with a large chorioretinal coloboma involving the optic nerve (white arrow in (d)) in a two-year-old girl with CHARGE syndrome. Axial T2WI shows left ocular microphthalmia with typical posterior coloboma (white arrow in (e)). Brain MR with a dedicated DRIVE sequence demonstrates bilateral cochleovestibular dysplasia typically observed in CHARGE syndrome (white arrows in (f)).

The term microphthalmia refers to a small ocular globe, with a corneal diameter of less than 10 mm and an anteroposterior diameter of less than 20 mm [21], resulting in an eye that is either 2 standard deviations below the age-adjusted mean [21]; or less than ⅔ of the normal diameter [10]; or below the 5^th^ percentile [10]. The terms microphthalmia and nanophthalmia are frequently considered synonyms. Nevertheless, some authors reserve the term nanophthalmia for severe cases in which: the axial length of the eye is inferior to 18 mm; microcornea coexists; and no systemic abnormalities are identifiable [7, 9, 21]. Microphthalmia prevalence at birth is around 14 per 100.000 live births and the combined birth prevalence of microphthalmia and anophthalmia is 30 per 100.000 population, which enforces the idea of them being two poles of a wide spectrum [19, 20]. Microphthalmia may be unilateral or bilateral and is divided into primary microphthalmia in case of genetic and syndromic origins, and secondary to different injuries [11, 21]. In both cases, microphthalmia may be isolated or syndromic if other facial or systemic anomalies are associated [7]. In case of later onset, microphthalmia is frequently isolated and unilateral, although it is a bilateral occurrence, generally [10, 33]. Other maternal and child factors may increase the risk of anophthalmia such as maternal age over 40, low birth weight, and premature birth [6]. Primary microphthalmia has been related to genetic mutations (SOX2, PAX6, OTX2, RAX, CHX10, FOXE3, and CRYBA4), chromosomal mutations (trisomy 13 and trisomy 18) [7, 10, 11, 21, 27, 34], and many syndromes such as CHARGE syndrome, Walker–Warburg syndrome, Dandy–Walker syndrome, Goldenhar syndrome, Lowe syndrome, and Fraser syndrome [10, 11]. Secondary microphthalmia has been related to retinopathy of prematurity (ROP), prenatal toxin exposure (alcohol), infections (TORCH), and other injuries seen in anophthalmia dissertation [7, 10, 11, 21, 34]. Microphthalmia may also be divided into two categories: simple or pure when the ocular globe is small but anatomically correct; and complex, when the eye is malformed [7, 10]. Taking into account that the posterior segment of microphthalmic eyes is more affected than the anterior, Weiss proposed that ocular growth after birth has great importance and speculated that small optic cup, low intraocular pressure, altered vitreous proteoglycans, and abnormal releases of growth factors as cofactors in the development of simple microphthalmia [7, 9, 21]. Inadequate production of secondary vitreous has been speculated to be the pathogenic mechanism of complex microphthalmia [35]. Complex microphthalmia is further classified into noncolobomatous and colobomatous forms, in relation to the presence of coloboma [7]. Patients' presentation ranges from vision preservation to vision loss depending on microphthalmia severity. Microphthalmia may be difficult to assess in prenatal US and MRI, especially in nonsevere forms. Postnatally, the US is commonly performed to assess the biometry of the globe. Simple microphthalmia may be detected at MRI as a smaller globe with normal density and signal, in a smaller orbit than normal (Figures 2(d)–2(f) and 3(a)–3(c)). As anophthalmia, microphthalmia should be differentiated from cryptophthalmos, cyclopia, and synophthalmia. Treatment of severe cases of microphthalmia is similar to anophthalmia treatment [31]. In bilateral microphthalmia with preserved retinal function, conservative management is suggested and consists of refracting the eyes and treating underlying amblyopia when present. If microphthalmia is unilateral, the treatment should be proportional to the severity of the disease, and the contralateral eye function should be carefully protected [21].

### 5.3. Coloboma

Coloboma represents a fissure or discontinuity potentially involving every ocular structure (choroid, pupil, eyelid, optic nerve, etc…), and it accounts for 2% of all congenital anomalies [36]. The most common type of coloboma results from failure of the choroidal fissure to close properly between the 5^th^ and the 7^th^ week of gestation and is classified as typical coloboma [7]. Ocular colobomas are often bilateral and small, without associated abnormalities. Nevertheless, coloboma has been associated with many genes, such as PAX6 and GDF6; and with many brain malformation syndromes such as Joubert syndrome, Aicardi syndrome, and Walker–Warburg syndrome [11, 37]. Clinical symptoms, MRI findings, and treatment are strictly related to the type of coloboma. The most common form of coloboma is located in the inferior temporal region of the iris and is often asymptomatic, cryptic to imaging [11], and treated with colored contact lenses or surgery (Figures 3(d)–3(f)). Two notable variants are Morning Glory syndrome and microphthalmia with cyst.

#### 5.3.1. Morning Glory Syndrome

Morning Glory syndrome is a coloboma of the optic disc that takes its name after a characteristic ophthalmoscopic appearance [11] resembling the flower Morning Glory [7]. Prevalence is unknown [38], but it has been most commonly found in females [39]. It has been speculated that cells of the future optic nerve remain undifferentiated for a long time and are therefore more sensitive to insults, similar to the cells at the margin of the embryonic fissure [40]. The Morning Glory syndrome has been associated with midline structure anomalies of the brain and skull as basilar encephaloceles and callosal agenesis [41]. The clinical presentation encompasses abnormality of retinal pigmentation, strabismus, and amblyopia [42, 43]. Visual acuity defects are nonprogressive and depend on the extent of optic nerve anomalies and may coexist with blind spots and visual field defects [44]. An increase in serous retinal detachment risk has been described. Diagnosis is clinical and relies on fundoscopy findings which consist of an enlarged optic disc with peripapillary pigmentations, a funnel-shaped deep excavation, a radiating pattern of retinal blood vessels, and a pale fluffy tuft of hyperplastic glial tissue overlying the optic disc [38].

**Figure 4:**
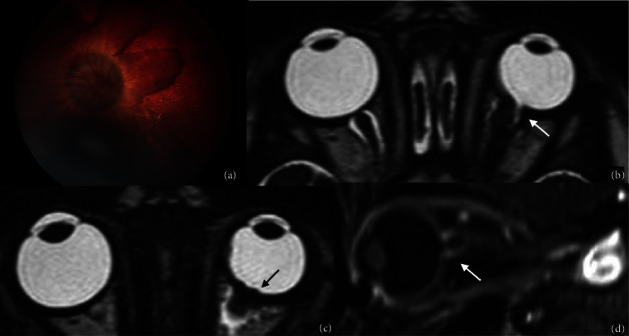
Morning Glory syndrome. RET-cam image in an eighteen-month-old boy with microphthalmia and Morning Glory malformation (a). MRI of the orbits confirms the malformation: axial high-resolution T2WI shows a funnel-shaped optic disc (white arrow in (b)) and the elevation of the retina (black arrow in (c)), whereas a sagittal postcontrast T1WI shows a discontinuous sclera profile (white arrow in (d)).

**Figure 5:**
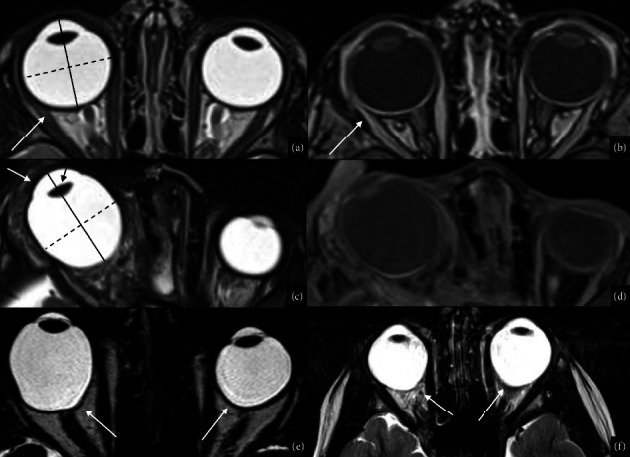
Macrophthalmia (macrophthalmus, buphthalmos, and axial myopia) and Staphyloma. MRI T2 FS WI (a) and postcontrast T1 FS WI (b) of a four-year-old boy with right macrophthalmus (white arrows in (a, b)). Macrophthalmus is characterized by a homogeneous increase in size (anteroposterior, continuous black line in (a); and transverse diameters, dotted black line in (a)) of the ocular globe. MRI T2 FS WI (c) and postcontrast T1 FS WI (d) of a one-year-old boy with right buphthalmos, secondary to congenital glaucoma. Buphthalmos is characterized by a prevalent enlargement of the anteroposterior diameter (continuous black line in (c)) compared to the transverse diameter (dotted black line in (c)). Increased diameter of the cornea and flattening of the ventral surface of the lens are also appreciable (white and black arrows, respectively). Axial DRIVE (e) of a ten-year-old girl affected by axial myopia in the left eye and bilateral staphyloma. Axial myopia is characterized by an increased size of the ocular globe and in particular an increased anteroposterior depth of the posterior segment of the eye. It may frequently be complicated by staphyloma, consisting of a posterior uveoscleral bulge, frequently appreciable on the nasal side of the optic disc (white arrows in (e)). MRI T2WI (f) of a six-year-old boy affected with bilateral staphyloma, which consists of posterior uveoscleral bulges, appreciable on the nasal side of the optic disc (white arrows in (f)).

Imaging may provide a better assessment of the pathology and help in the differential diagnosis. In particular, MRI is the Imaging modality of choice and shows a funnel-shaped morphologic pattern of the optic disc with elevation and hyperintensity of the adjacent retinal surface on T1WI, coexisting with discontinuity of the uveoscleral coat at the optic nerve insertion. Within the distal intraorbital segment of the ipsilateral optic nerve, an abnormal tissue, showing contrast enhancement on T1WI after gadolinium administration, and effacement of the subarachnoid space, better visible on T2WI, may be detected. In the optic sheath, at the same level, a fat deposit is evident [43] (Figure 4). The main differential diagnosis is staphyloma, consisting of scleral-uveal coats stretching with a uveal protrusion, typically occurring temporally from the optic disc, and optic nerve coloboma [45] (Figure 5(f)). Therapy consists of the treatment of the presenting signs and symptoms. Particularly, amblyopia is usually treated by patching or atropine administration to the contralateral eye [46], strabismus may be corrected with surgery [47], and serous retinal detachments do not usually require treatment but follow-up controls [38].

#### 5.3.2. Microphthalmia with Cyst

**Figure 6:**
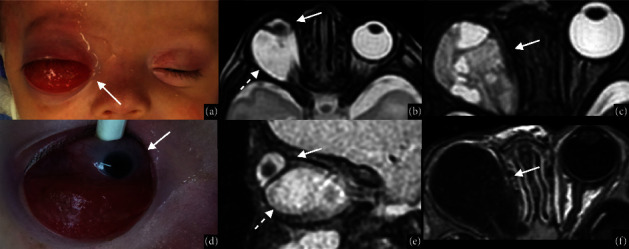
Coloboma/microphthalmia with cyst vs congenital cystic eye. A thirteen-day-old girl with suspected right anophthalmia with cyst (white arrow in (a)) underwent an MRI whose axial (b) and sagittal (e) T2WI sequences showed the presence of severe microphthalmia (white arrows in (b, e)) with associated cyst (dotted white arrow in (b, e)), that was subsequently confirmed during clinical observation under sedation (d). Axial high-resolution T2WI (c) and axial postcontrast T1 (f) in a two-month-old boy affected by congenital cystic eye show a multilobulated cystic formation without an ocular globe (white arrow in (c, f)).

It is an extremely rare and usually unilateral microphthalmia that might also be referred to as coloboma with cyst and represents a severe malformation caused by the massive proliferation of the retinal inner layer at the margins of the unclosed choroidal fissure and its outward protrusion forming a uni- or multilobulated cystic mass [7, 10, 48]. Cystic walls may fuse with the wall of the globe and create a continuity between the cyst and the vitreous. The size of the ocular globe and the cyst vary: a massive extrusion of vitreous humor into the cyst may result in a small ocular globe and a huge cyst, which causes ocular proptosis and bulging of the lower lid [10, 11]. Patients may present with multiple signs and symptoms ranging from mild vision impairment to complete blindness [49]. Although the diagnosis is mostly clinical [49], imaging is crucial to characterize the eye, the cyst, and the continuity between the ocular globe and the cyst [50]. The key feature for a correct differential diagnosis is the presence of the crystalline lens in the ocular globe [7, 11]. US shows an orbital echo-lucent cyst indenting the globe, while MRI guarantees a more detailed picture of the cystic lesion, which generally appears hyperintense on T2WI, and demonstrates its association with the microphthalmic globe and other orbital structures [21, 51]. Associated brain anomalies should always be investigated with MRI [7] (Figures 6(a), 6(b), 6(d), and 6(e)). Microphthalmia with cyst should be differentiated from the congenital cystic eye which is secondary to failure of the optic disc to invaginate [52] (Figures 6(c) and 6(f)). Other differential diagnoses are encephaloceles, dermoid and epidermoid cysts, and necrotic teratomas [49]. There is no widely accepted treatment strategy for patients with microphthalmos and orbital cysts [49]. Chaundhry et al. [49] suggest a tailored therapy related to the severity of microphthalmos and the size/enlarging properties of the cyst [52].

## 6. Macrophthalmia

Macrophthalmia is characterized by an increased globe size and includes many differentials in relation to the type of globe enlargement: macrophthalmus, buphthalmos, and axial myopia. Imaging, and in particular MRI, is useful for differential diagnosis and detection of associated cerebral disorders, such as syndromic or infective associations [53].

### 6.1. Macrophthalmus

Macrophthalmus presents with an enlargement of all dimensions of the eye and is not related to congenital glaucoma (Figures 5(a) and 5(b)). It is usually diagnosed in patients with neurofibromatosis, although in these cases it may be associated with buphthalmos secondary to congenital glaucoma [53].

### 6.2. Buphthalmos

Buphthalmos has a prevalence of 1 : 30000 births [54], and in 80% of cases is bilateral [53–55]. The most common cause of buphthalmos is congenital glaucoma which may be isolated or syndromic and occurs at birth or within the first 3 years [11, 56]. The enlargement of the globe coexists with an increased diameter of the cornea (>12 mm) and rupture of Descemet's membrane (Haab's striae) [54]. MRI shows flattening of the ventral surface of the lens [10], thinning and low attenuation of the sclera and the choroid [53], and optic disc cupping [54] (Figures 5(c) and 5(d)).

### 6.3. Axial Myopia

Axial myopia is generally related to collagen disorders such as Ehlers–Danlos syndrome [10] and presents with an anteroposterior enlargement of the globe, particularly of the posterior chamber of the globe, with normal cornea [55] (Figure 5(e)). Severe axial myopia is often associated with scleral ectasia resulting in staphyloma [55] (Figure 5(f)) and attenuation and local bulging of the uveal-scleral rim, amblyopia and secondary retinal degeneration [53].

## 7. Persistent Hyperplastic Primary Vitreous

Persistent hyperplastic primary vitreous (PHPV) is a rare congenital ocular malformation occurring when the regression of the embryonic hyaloid vascular system fails. Most cases of PHPV are sporadic, yet it can be inherited through an autosomal dominant or recessive transmission. Genes responsible for PHPV are still not completely known; however, in animal models defects in the Wnt signaling pathway or apoptosis contribute to the development of this malformation [57]. PHPV is usually unilateral, but bilateral cases have been described, and when bilateral, it is usually associated with other diseases such as Norrie's disease, characterized by PHPV, deafness, mental retardation, and absence of the retinal ganglion cells, and Warburg disease, which is an autosomal recessive syndrome characterized by PHPV, hydrocephalus, lissencephaly, and mental retardation, and trisomy 13 [57–59].

The most common clinical sign is leukocoria, presenting during the first months after birth. Other clinical manifestations are microphthalmos, shallow anterior chamber, congenital cataract, and strabismus [58]. Secondary closed-angle glaucoma may represent a severe complication [58].

The primary vitreous appears around the 7^th^ week of gestation life, lays between the lens and the retina, and contains the hyaloid vessels and fibrillar ectodermal tissues. Between the 5^th^ to the 8^th^ month of gestation, it gradually disappears and is replaced by the secondary and final vitreous. If the embryonic hyaloid vascular system fails to regress by the time of birth the PHPV occurs. Since the anterior and posterior hyaloid vascular systems regress independently, the patient may show a pure posterior type, a pure anterior type, or a more frequent combined type [58].

Ocular US shows an echogenic mass posterior to the lens and a hyperechoic band extending from the posterior surface of the mass to the posterior pole of the globe. Eco-color-Doppler demonstrates the presence of flow within the band representing the persistence of the hyaloid artery in the Cloquet canal. MRI is superior since it may differentiate posterior and anterior forms [58, 60].

**Figure 7:**
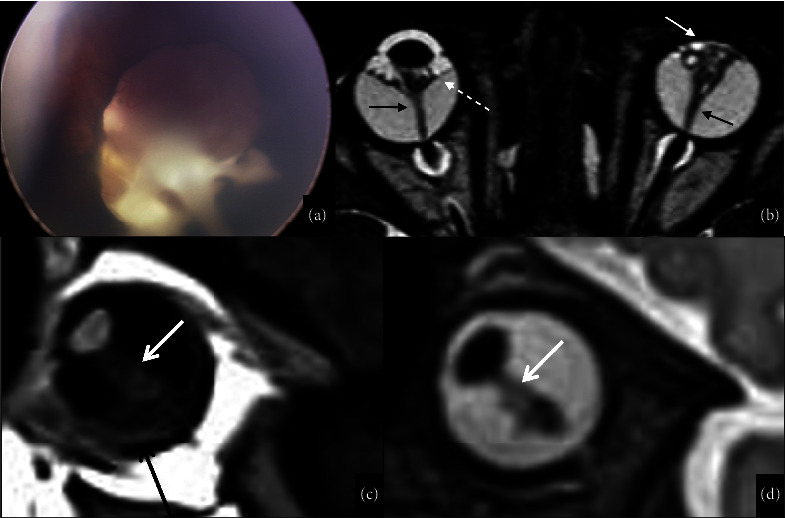
Persistent hyperplastic primary vitreous vs retinopathy of the prematurity. Persistent hyperplastic primary vitreous (a, b). The fundus examination of a one-year-old boy affected by Norrie's disease shows persistent hyperplastic primary vitreous with the persistence of the hyaloid vessels and posterior membrane in the vitreous (a). A high-resolution T2WI sequence confirms the malformation and shows retinal detachment in the right eye (dotted white arrow in (b)) and bilateral presence of the so-called “martini glass sign” characterized by a triangular-shaped retrolental mass connected to the head of the optic nerve by a tubular image along the Cloquet canal (black arrows in (b)). In the left eye, there is a collapsed anterior chamber (white arrow in (b)) suggesting the coexistence of the anterior form of PHPV. Retinopathy of the prematurity in a newborn infant of 91 days, born at 27 weeks of gestational age and undergoing oxygen supplementation (c, d). Sagittal T2WI (c) and sagittal postcontrast T1WI (d) show retinal detachment appearing as folded membranes (white arrows in (c, d) with subretinal space fluid). A minimal hemorrhagic component is visible on the T1WI (black arrow in (c)).

The posterior type is characterized by microphthalmos and the presence of a triangular-shaped retrolental mass appearing hypointense on T1WI and T2WI and enhancing after contrast administration. It represents the persistent fibrovascular tissue connected to the head of the optic nerve by the hyaloid artery running through the Cloquet canal. This finding is known as the “martini glass” sign [61], namely the presence of intravitreous hemorrhage due to the rich fibrovascular tissue in PHPV [59] and of retinal detachment, due to the traction of the retina by the fibrovascular mass, with possible fluid-fluid level corresponding to subretinal hemorrhage [58, 60] (Figures 7(a) and 7(b)).

In the anterior form, MRI shows a shallow or collapsed anterior chamber, enlarged vessels in the iris, and the presence of a retrolental-enhanced vascular membrane [62].

PHPV needs to be differentiated from other causes of leukocoria such as retinoblastoma, Coats disease, and ROP. The presence of microphthalmos and the absence of calcifications, detectable with CT or with MRI gradient-echo (GE) or susceptibility WI (SWI) sequences, are two radiological features that lead to the diagnosis of PHPV rather than retinoblastoma. Coats disease and ROP are different disorders and will be discussed in the next paragraphs [59].

Treatment goals are to save vision, correct amblyopia, and prevent/correct glaucoma. An early surgical approach, associated with the implantation of an intraocular lens for optical correction, and aggressive antiamblyopic therapy, can result in positive outcomes [62, 63].

## 8. Retinopathy of Prematurity

ROP is considered a disorder of retinal blood vessel development in preterm infants of low birth weight [64]. It was estimated that a global number of 184.700 children and 14,9 million preterm neonates developed ROP in the year 2010, and the incidence of ROP was 68% among neonates weighting <1.251 g [65, 66].

According to the revised two-phase hypothesis of ROP development, the paucity of blood vessels in the immature retina creates a peripheral avascular area (phase 1), that promotes intravitreal neovascularization in a later stage (phase 2) [64]. Lack of vascularization can lead to retinal ischemia, release of proangiogenesis factors, and neovascularization, eventually progressing to vitreous hemorrhage, retinal detachment, and blindness [67]. In many patients, vitreous neovascularization spontaneously regresses, with the only finding of retinal detachment. In some patients, the disease can progress to the chronic stage, characterized by fibrosis and scarring [67].

The two major risks for ROP are low birth weight and gestational age. The use of supplemental oxygen is another major risk factor for ROP because fluctuating high oxygen pressure can inhibit the production of proangiogenesis factors and interfere with developmental angiogenesis [64]. Recent studies suggested that ROP may be part of a wider neuro-vascular developmental disorder, involving structural abnormalities of the central nervous system, with long-term neuro-cognitive manifestations [68].

Screening is pivotal to identifying ROP and should begin within 4 weeks postpartum. A dilated fundus exam is mandatory in all preterm neonates born ≤30 weeks and infants with birth weight ≤1500 g. Clinical presentation is often related to vision impairment ranging from mild to complete blindness [65, 69, 70].

The International Classification of Retinopathy of Prematurity divides the retina into three zones and scores the severity of ROP in 5 stages depending on the specific retinal and vascular pattern at the border of the vascular and avascular retina [65, 71]. Imaging of ROP is focused on disease complications. The US can demonstrate bilateral microphthalmia and retinal detachment. Similar findings can be found on CT and MRI. MRI displays retinal chronic hemorrhages with variable signal intensity and retinal detachment, visible as a posterior chamber mass due to the opposition of the detached retinal leaves [59] (Figures 7(c) and 7(d)). Advanced MRI analysis at an older age may reveal reduced brain volumes [72] and microstructural abnormalities of white matter bundles, including the optic radiations on diffusion-tensor imaging (DTI) [73].

Early treatment consists of the administration of antivascular endothelial growth factor (VEGF), laser photocoagulation, and cryotherapy.

## 9. Coats Disease

Coats disease is a usually unilateral, retinal vascular disorder characterized by retinal telangiectasia and intraretinal and subretinal exudation that can lead to progressive retinal detachment and blindness [74, 75]. There are no ethnic differences, but there is a strong male predominance [76]. The incidence peak is between 6 and 8 years, ranging from 6 months to 70 years [75].

In Coats disease, there are telangiectasic and other vascular abnormalities with progressive retinal edema and the presence of lipidic exudate that is typical of the disease. Progressive subretinal leakage leads to retinal detachment and loss of vision [77].

There are five stages with increasing severity: Stage 1, asymptomatic retinal telangiectasia; Stage 2, telangiectasia with exudation; Stage 3, subtotal exudative retinal detachment; Stage 4, total retinal detachment; Stage 5, a secondary complication of retinal detachment [74–76].

Coats disease is usually an isolated disorder. Bilateral eye involvement in association with leukodystrophy, small brain cysts, and brain calcification involving basal ganglia and the deep white matter has been described as Coats plus syndrome or CRMCC syndrome (cerebro-retinal microangiopathy with calcification and cyst) [78, 79].

Patients with Coats disease usually present unilateral vision loss, strabismus, and xanthocoria [74].

Ophthalmoscopy shows saccular vascular aneurysms with tortuous, telangiectatic capillaries, exudate, and retinal hemorrhage [77].

In the early stage, imaging is often normal, while in the advanced stage retinal detachment is clear. The US allows globe assessment, yet it presents limitations in the differential diagnosis between Coats disease and retinoblastoma. To exclude Coats plus syndrome, an MRI examination of the brain should be performed in any patient with Coats disease, especially in the case of bilateral Coats disease [75, 79].

**Figure 8:**
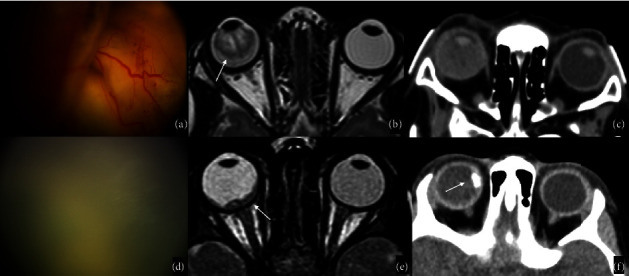
Coats Disease vs Retinoblastoma. Coats Disease (a–c). Fundus examination of a two-year-old boy presenting with exudative retinal detachment (a). An axial T2WI of the orbits confirms the presence of complete retinal detachment with vitreous hemorrhage (white arrow in (b)), while CT demonstrates the absence of ocular calcifications. Findings are consistent with Coats Disease. Retinoblastoma (d–f). Fundus examination of a three-year-old boy with diffuse vitreous seeding involving the whole vitreous cavity of the right eye. An axial high-resolution T2WI sequence shows diffuse irregular retinal thickening in the right eye (white arrow in (e)) associated with a focal calcification on CT (white arrow in (f)). Findings are consistent with the clinical suspicion of Retinoblastoma.

On MRI, the subretinal fluid exudate presents high signal intensity on both T1WI and T2WI due to its fat content, yet a heterogeneous signal may be referred to as hemorrhage or fibrosis. The subretinal space does not enhance after gadolinium administration, but the linear enhancement of the choroid converging to the optic disc may highlight the subretinal space at the border between the exudate and the remaining vitreous [75, 77, 80] (Figures 8(a)–8(c)). This finding is typical of Coats disease, assessing contrast-enhancement MRI as the imaging of choice for the diagnosis of Coats disease [75]. Calcifications are normally absent, yet in the advanced stages, calcified submacular nodules and bone formation along the vessels can be seen on GE or SWI sequences [81]. MR spectroscopy detects a large lipid peak, probably related to the presence of lipoproteinaceous material [82, 83].

The main differential diagnosis is retinoblastoma, which is usually hypointense on T1WI and hyperintense on T2WI with a mass-like enhancement after contrast administration. Calcifications are much more common in retinoblastoma and are evident on CT scans [75] (Figures 8(d)–8(f)).

The subretinal fluid heterogeneous appearance, the normal morphology of the lens, the absence of retrolental mass, and Cloquet's canal outline allow the differential diagnosis with PHPV [75, 84].

Treatment is controversial and includes surgical repair of retinal detachment, cryotherapy, and laser photocoagulation.

## 10. Congenital Defects of the Crystalline Lens

Crystalline lens congenital defects are extremely varied disorders, a leading one being congenital cataracts [85]. Ocular examination and US are generally optimal methods to diagnose lens abnormalities, which contribute to 9.2% of blindness [86], although the frequent association with other ocular disorders, infective diseases, and syndromes may benefit of MRI evaluation [85].

### 10.1. Aphakia

Aphakia is the absence of the crystalline lens in the eye and arises from the failure of lens induction from the surface ectoderm (primary aphakia) or the abortion of lens development with spontaneous resorption (secondary aphakia, more common) [87, 88] (Figures 3(b)). Congenital primary aphakia, whose prevalence ranges from 1 to 5/10.000 births [89] is caused by variants in the FOXE3 gene and most cases are autosomal recessive.

**Figure 9:**
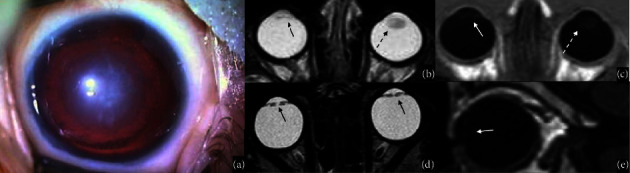
Congenital cataract and pseudophakia. Congenital cataract in a one-month-old girl (a). MRI axial T2WI (b) and postcontrast axial FS T1WI (c) of a three-month-old girl with bilateral cataracts. In the left eye, the cataract appears as a thickened crystalline lens, hyperintense both on T2WI and T1WI (black dotted arrow in (b) white dotted arrow in (c)). In the right eye, a cataract was treated, and an intraocular lens was placed: this apparent absence of the lens is called pseudophakia (black arrow in (b) white arrow in (c)). Axial DRIVE (d) and sagittal postcontrast T1WI (e) of a two-year-old patient affected by bilateral congenital cataract, secondary to galactosemia. In both eyes, the cataracts were treated and intraocular lenses were implanted, hence showing a pseudophakic appearance (black arrows in (d) white arrow in (e)).

Differential diagnosis is with pseudophakia, in which the aphakic appearance is related to the insertion of intraocular lens implantation (Figure 9(d)).

The treatment of choice is the surgical insertion of an intraocular lens implant.

### 10.2. Congenital Ectopia Lentis

Congenital ectopia lentis (CEL) is a congenital zonular disease that leads to displacement of the lens from its natural position, with a prevalence of 6/100.000. It usually occurs bilaterally, and it is often associated with inherited connective tissue disorders such as Marfan syndrome [90, 91].

**Figure 10:**
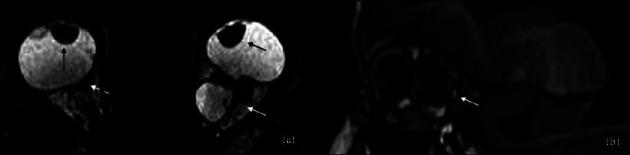
Congenital ectopia lentis and complex eyes malformations in an eighteen-month-old boy. Axial high-resolution T2WI (a) and sagittal T1WI (b) show bilateral dysmorphic and displaced lenses (black arrows in (a)), right coloboma (dotted arrow in (a)) and left microphthalmia/coloboma with cyst, which is characterized by a multiloculated cystic mass (white arrows in (a) and (b)) extending in the left orbital conus.

CEL may cause a high refractive error, myopia, astigmatism, glaucoma, retinal detachment, diplopia, and cataracts [91–93]. At the ocular examination, an aphakic crescent sign is pathognomonic for zonular disruption and subluxation of the lens and may be appreciated if the pupil is dilated [93]. MRI shows an ectopic position of the lens in the anterior chamber or the vitreous (Figure 10).

Surgery for intraocular lens implantation includes the implantation of anterior chamber intraocular lenses, or of scleral-fixated or iris-fixated posterior chamber intraocular lenses. Recently, the use of capsular tension rings has also been described [94].

### 10.3. Congenital Cataract

Cataract refers to the opacification of the crystalline lens and represents the most common treatable cause of childhood blindness, with a prevalence of 1,03/10.000 children [95]. Causative mechanisms range from idiopathic (generally unilateral) to hereditary (mostly autosomal dominant), metabolic (galactosemia, hypoglycemia), syndromic (Down's, Lowe's, Alport's, Norrie's), infectious (TORCH), traumatic, secondary (uveitis, retinoblastoma), and iatrogenic (postretinal detachment surgery; postlaser in ROP) [96–102].

Small children present with eye squinting, microphthalmos, buphthalmos, squeezing of the eye, and nystagmus, while older children have difficulty viewing distant objects and bringing things close to their faces to facilitate vision [103].

Cataract screening by searching for the red reflex with the ophthalmoscope is an essential component of the exam and the absence/anomaly of the red reflex indicates urgent referral [85]. US may rule out retinal detachment, fundal coloboma, and retinoblastoma, and in case of unilateral cataracts may reveal persistent fetal vasculature thanks to Color Doppler. MRI is crucial in the suspicion of PHPV and/or encephalic abnormalities. Crystalline may present as a hyperintense on T2WI, thickened crystalline lens or it may appear normal in size and signal characteristics [104] (Figures 9(a)–9(c)).

The treatment of choice is the surgical extraction of the lens nucleus from its capsule and the insertion of an intraocular lens implant.

## 11. Conclusions

Malformations of the eye represent a various group of pathologies with different etiologies and prognoses. The knowledge of different disorders together with a dedicated ocular imaging and brain MRI to rule out syndromic conditions, are fundamental for the radiologist to frame ocular malformations.

We believe that the multidisciplinary team discussion with contributions from ophthalmologists, surgeons, and radiologists is crucial to ensure early diagnosis and tailored therapy and to improve patients' quality of life. It is pivotal to promote the centralization of cases to be analyzed during multidisciplinary meetings to gain expertise on the specific features and characteristics of each ocular malformation to guarantee the best assistance to patients. This may be favored by technical improvements in the diagnostic, clinical, and surgical fields. Moreover, peculiar cases may be the perfect material for supporting medical education and formation to spread knowledge among clinicians, radiologists, and surgeons.

## Figures and Tables

**Table 1:** Proposed orbital MRI protocol and brain MRI protocol to detect associated brain malformations [17–19].

Orbit		Brain	
Scanner and coils			
(i) 1.5-T preferentially with one or two small surface coils (diameter <5 cm)		(i) 1.5-T or 3.0-T with 32- or 64-channel head coil	
(ii) 3.0-T with 32- or 64-channel head coil			

Sequences^+^			
*Precontrast*	*Postcontrast*	*Precontrast*	*Postcontrast (optional)*

(i) Axial^∧^T2WI TSE FS ≤3 mm	(i) Axial^∧^T1WI TSE FS or without FS but with subtraction ≤3 mm	(i) Coronal T2WI TSE ≤4 mm	(i) 3D T1WI 1 mm
(ii) 3D high-resolution heavily T2WI (SPACE/CISS/FIESTA-C/VISTA/ Cube) ≤0.6 mm	(ii) SagittalOblique° T1WI TSE FS or without FS but with subtraction (optional) ≤3 mm	(ii) Axial T2WI TSE ≤4 mm	
(iii) Coronal^§^T2WI TSE FS ≤3 mm		(iii) Axial FLAIR ≤4 mm	
(iv) Axial^∧^ T1WI TSE ≤3 mm		(iv) 3D T1WI 1 mm	
(v) Coronal T1 WI TSE		(v) Axial DWI ≤4 mm	
(vi) Sagittal Oblique° T1WI TSE (optional) ≤2 mm			
(vii) SWI^*∗*^ (optional)			

FS: fat sat, WI: weighted image, SPACE: sampling perfection with application-optimized contrasts by using fip angle evolution, CISS: constructive interference in steady state, FIESTA fast imaging employing steady state acquisition, VISTA volume isotropic turbo spin echo acquisition. ^+^2D in-plane sequences for the orbits should be acquired with a minimal slice gap and high acquisition matrices of 448 to 512, with a field-of-view of approximately 18 to 20 cm, obtaining a 0.4 mm in-plane resolution. ^Axial scans should cover the area between the hard palate and approximately 1 cm above the orbits. ^§^Coronal scans should extend from the lens to the midpons. °Sagittal oblique scans should be aligned along the course of the optic nerve. ^*∗*^For the assessment of ocular calcifications.

**Table 2:** Main epidemiological and clinical features of ocular malformations and their related diagnostic pathway and management.

Congenital ocular malformations		Prevalence	Clinical features and symptoms	Diagnosis	Management
Anophthalmia		3 per 100.000 live births	Absence of the eye → blindness	Clinical MRI is useful in syndromic patients	Simultaneous expansion of the eyelids, socket and orbital bones followed by dermis-fat grafts and orbital implants

Microphthalmia		14 per 100.000 live births	Small eye → range from preservation to vision loss	Clinical MRI is useful in syndromic patients	Severe cases → similar to anophthalmia treatment Bilateral microphthalmia with preserved retinal function → optimal eye refraction + amblyopia correction Unilateral microphthalmia → optimal eye refraction + protection of contralateral eye function
Coloboma	Morning Glory	Unknown, most common in females	Abnormality of retinal pigmentation, strabismus, and amblyopia	Ophthalmoscopy MRI is crucial to exclude midline structures anomaly of the brain and skull	Treatment of the presenting signs and symptoms Amblyopia → patching or atropine administration to the contralateral eye Strabismus → surgery serous retinal detachments → follow-up controls
	Microphthalmia with Cyst	Extremely rare	Range from mild vision impairment to complete blindness	Mostly clinical US/MRI are crucial to → characterize the eye, the cyst, and a continuity between the ocular globe and the cyst → exclude associated brain anomalies	Tailored therapy related to the severity of microphthalmos and the size/enlarging properties of the cyst

Macrophthalmia		1 per 100.000 live births (buphthalmos)	Glaucoma, myopia, amblyopia, and secondary retinal degeneration	Clinical, ophthalmoscopy, and imaging brain MRI is crucial for associated syndromes (es. neurofibromatosis in macrophthalmus)	Treatment of the presenting signs and symptoms

Persistent hyperplastic primary vitreous		Rare	Leukocoria (most common), microphthalmos, shallow anterior chamber, congenital cataract and strabismus, and secondary closed-angle glaucoma	Clinical, ophthalmoscopy, and imaging	Early surgical approach to implant an intraocular lens Antiamblyopic therapy Prevention/treatment of glaucoma

Retinopathy of prematurity		More common in preterms with low weight at birth incidence of 68% among neonates weighting <1.251 g	Range from mild vision impairment to complete blindness	Clinical, ophthalmoscopy	Antivascular endothelial growth factor (VEGF) Laser photocoagulation, Cryotherapy

Coats disease		Strong male predominance	Vision impairment/loss, strabismus and xanthocoria	Clinical, ophthalmoscopy, and imaging brain MRI is crucial for associated syndromes (Coats plus syndrome)	Treatment is controversial and includes surgical repair of retinal detachment, cryotherapy, and laser photocoagulation

Congenital defects of the crystalline lens	Aphakia	1 to 5 per 100.000 live births	Vision impairment	Clinical, ophthalmoscopy	Surgery → insertion of an intraocular lens implant
	Congenital Ectopia Lentis	6 per 100.000 live births	High refractive error, myopia, astigmatism, glaucoma, retinal detachment, diplopia, and cataract	Clinical, ophthalmoscopy	Surgery → intraocular lens implantation (anterior chamber intraocular lenses, or scleral-fixated/iris-fixated posterior chamber intraocular lenses)
	Congenital Cataract	1 per 100.000 live births	Small children → ye squinting, microphthalmos, and buphthalmos, squeezing of the eye and nystagmus Older children → refer difficulty in viewing distant objects and bringing things close to their face to facilitate vision	Clinical, ophthalmoscopy US → concomitant eye malformations/pathologies Brain MRI → associated syndromes	Surgery → extraction of the lens nucleus from its capsule + insertion of an intraocular lens implant

## Abbreviations

MRI: Magnetic resonance imaging
WI: Weighted imaging
DTI: Diffusion-tensor imaging
US: Ultrasound
CT: Computed tomography
MAC: Microphthalmia, anophthalmia, and coloboma
TORCH: Toxoplasmosis, other, rubeola, cytomegalovirus, herpes
CHARGE: Coloboma, heart disease, atresia of the choanae, retarded growth and mental development, genital anomalies, and ear malformations
ROP: Retinopathy of the premature
PHPV: Persistent hyperplastic primary vitreous
CRMCC: Cerebro-retinal microangiopathy with calcification and cyst
VEGF: Antivascular endothelial growth factor
CEL: Congenital ectopia lentis
GE: Gradient-echo
SWI: Susceptibility WI.
